# Does the presence of an implanted cardiac device adversely affect the image quality of clinically indicated magnetic resonance imaging at 1.5t?

**DOI:** 10.1186/1532-429X-17-S1-P241

**Published:** 2015-02-03

**Authors:** Kanae Mukai, Heather Costa, Robert J Russo

**Affiliations:** Cardiology, Scripps Clinic, La Jolla, CA USA; Scripps Clinic Green Hospital, La Jolla, CA USA; Scripps Clinic, La Jolla, CA USA

## Background

The purpose of this study was to determine the incidence of significant device-related imaging artifacts in patients with a pacemaker or an implanted cardioverter-defibrillator (ICD) undergoing clinically-indicated thoracic and non-thoracic MRI at 1.5T.

## Methods

Radiology reports were reviewed for the presence of either a cardiac device-related artifact on MRI that was identified but did not affect study interpretation, or an artifact that adversely affected image quality, with or without the suggested requirement for an alternate imaging modality.

## Results

Between November 2003 and February 2014, 1000 consecutive clinically indicated MRI examinations were performed on 569 patients with an implanted cardiac device at a single center. Scan locations included: Brain 315, spine 358, extremities 82, abdomen/pelvis 56, cardiac 24, shoulder 32 (19 left and 13 right), and orbit/face/head/neck 133. Cardiac device-related imaging artifacts were reported in 1.0% of non-cardiac scans (11 of 976) which included 7 left shoulders scans with an ipsilateral device (4 ICDs/3 pacemakers), 1 right upper extremity scan with a left-sided pacemaker with metallic artifact (and a prosthetic right humeral head), 1 cervical spine scan with a left-sided pacemaker, 1 neck scan with a right-sided ICD, and 1 orbit/face/neck scan with a left-sided ICD. No significant imaging artifacts were noted in 10 of the 16 left shoulder MRI studies (63%) which included 1 contralateral and 9 ipsilateral cardiac device implant positions. Overall, artifacts were most pronounced on gradient echo images. For cardiac scans, 14 of 24 studies (58%) had a device-related artifact which precluded full or partial study interpretation (7 pacemakers/7 ICDs), and all had a left-sided device implant. Cardiac 3-D volumetric assessment could not be performed in 8 studies, delayed-enhancement imaging was not possible in 1 study, and image quality was severely limited in 3 additional studies. Two cardiac studies contained an artifact that adversely affected image quality with the requirement for an alternate imaging modality: one standard cardiac study and one cardiac study with aortic angiography.

## Conclusions

The presence of an implanted pacemaker or ICD rarely affected the image quality of clinically indicated non-cardiac MRI at 1.5T. Non-cardiac artifacts were most frequently present on left shoulder MRI studies with an ipsilateral device implant.

